# The topology of connections between rat prefrontal, motor and sensory cortices

**DOI:** 10.3389/fnsys.2014.00177

**Published:** 2014-09-17

**Authors:** Stacey A. Bedwell, E. Ellen Billett, Jonathan J. Crofts, Chris J. Tinsley

**Affiliations:** School of Science and Technology, Nottingham Trent UniversityNottingham, UK

**Keywords:** prefrontal cortex, sensory-motor cortex, connections, organization

## Abstract

The connections of prefrontal cortex (PFC) were investigated in the rat brain to determine the order and location of input and output connections to motor and somatosensory cortex. Retrograde (100 nl Fluoro-Gold) and anterograde (100 nl Biotinylated Dextran Amines, BDA; Fluorescein and Texas Red) neuronanatomical tracers were injected into the subdivisions of the PFC (prelimbic, ventral orbital, ventrolateral orbital, dorsolateral orbital) and their projections studied. We found clear evidence for organized input projections from the motor and somatosensory cortices to the PFC, with distinct areas of motor and cingulate cortex projecting in an ordered arrangement to the subdivisions of PFC. As injection location of retrograde tracer was moved from medial to lateral in PFC, we observed an ordered arrangement of projections occurring in sensory-motor cortex. There was a significant effect of retrograde injection location on the position of labelled cells occurring in sensory-motor cortex (dorsoventral, anterior-posterior and mediolateral axes* p* < 0.001). The arrangement of output projections from PFC also displayed a significant ordered projection to sensory-motor cortex (dorsoventral *p* < 0.001, anterior-posterior *p* = 0.002 and mediolateral axes *p* < 0.001). Statistical analysis also showed that the locations of input and output labels vary with respect to one another (in the dorsal-ventral and medial-lateral axes, *p* < 0.001). Taken together, the findings show that regions of PFC display an ordered arrangement of connections with sensory-motor cortex, with clear laminar organization of input connections. These results also show that input and output connections to PFC are not located in exactly the same sites and reveal a circuit between sensory-motor and PFC.

## Introduction

Prefrontal cortex (PFC) has been strongly associated with executive function, temporal ordering, cognitive processes and autonomic functions (Kolb, 1984; Neafsey, 1990; Alvarez and Emory, 2006; Schoenbaum and Esber, 2010). Prefrontal cortex has also been implicated in a number of neurological abnormalities such as autism and psychosis (Goldman-Rakic, 1991; Perlstein et al., 2001; Courchesne et al., 2011). Rodent PFC contains medial PFC (mPFC), orbital and agranular insular regions (Van De Werd and Uylings, 2008) which are thought to be functionally distinct. Dorsal mPFC has functional links to motor cortex and is involved in motor and temporal processing (Narayanan and Laubach, 2006; Vertes, 2006; Kim et al., 2013). Ventral mPFC has a functional role in cognitive and emotional processing (Frysztak and Neafsey, 1994; Vertes, 2006). Rat orbital cortex has been proposed to be involved in associative learning and making predictions about the external environment (Schoenbaum and Roesch, 2005; Schoenbaum and Esber, 2010). Agranular insular cortex has a greater functional role in the processing of sensory information including gustation (Gallagher et al., 1999; Fujita et al., 2011).

Despite the advances in our understanding of PFC function in the rat, the precise neuronal circuitry of PFC regions remains largely undefined, meaning the functional connectivity cannot yet be fully understood. Topographic organization has been described as a hallmark feature of cortical organization among vertebrates (Thivierge and Marcus, 2007) and is widely regarded as a necessary feature for complex brain function. Ordered structural and physiological organization has been described for several regions of cerebral cortex, including motor, sensory, auditory, visual and entorhinal cortex (Woolsey, 1967; Welker, 1971; Hafting et al., 2005). Connectivity studies of these regions have often indicated that ordered functional organization is based upon an underlying topographically ordered organization of anatomical projections (Porter and White, 1983; Henry and Catania, 2006; Aronoff et al., 2010).

Recent studies into PFC connectivity broadly indicate an ordered structural organization (Hoover and Vertes, 2011; Kondo and Witter, 2014). Kondo and Witter (2014) described a topographic organization of the projection from orbitofrontal cortex to the parahippocampal region in rats. They demonstrated an ordered arrangement of output connections from PFC sub-regions (medial orbital (MO), ventral orbital (VO), lateral orbital (LO)) to areas of perirhinal, postrhinal and entorhinal cortex. This ordered arrangement was reported only for the output projection. Parallel arrangement of input and output connections, resulting in reciprocal connections is a widely accepted occurrence in cortical organization, and has been described in multiple regions, including perirhinal, postrhinal, entorhinal, piriform, frontal, insular, temporal, cingulate, parietal and areas of occipital cortex (Canto et al., 2008; Agster and Burwell, 2009).

Studies of rat PFC outputs have shown that they also project widely to subcortical (such as the thalamus, striatum, brainstem and amygdala) and cortical targets (such as perirhinal and entorhinal cortex) (Vertes, 2004; Gabbott et al., 2005; Hoover and Vertes, 2007). Such studies have also shown that rat PFC displays gross-level topologically organized connections in rats. Ordered projections from lateral and posterior PFC regions to the posterior cingulate area have been reported (Olson and Musil, 1992). Recent anatomical studies report a topologically organized projection from rat lateral PFC to perirhinal cortex and area TE (Hoover and Vertes, 2011). Tracer studies have demonstrated a broad medial-lateral topographic organization of connections from PFC sub-regions to sub-cortical structures (Berendse et al., 1992; Schilman et al., 2008): a medial-lateral shift in injection sites in PFC produced a corresponding medial-lateral shift in anterogradely labelled projections in striatum and caudate putamen. However, this ordered arrangement has only been reported in subcortical PFC projections, and is described only between cytoarchitecturally distinct PFC sub-regions.

In order to clearly establish the nature of physiological organization within PFC, it is necessary to first gain a more detailed picture of the underlying anatomical organization. Anterograde tract tracing findings have revealed spatially ordered projections from the posterior PFC (mPFC), both within and across different cytoarchitectural areas (Sesack et al., 1989). Further to this, retrograde tracers reveal projections of MO and VO cortices in the rat (Berendse et al., 1992; Schilman et al., 2008). The prominent cortical targets (orbital fields) of MO and VO were found to be adjacent (Berendse et al., 1992), demonstrating a topographic organization of MO and VO projections to medial dorsal striatum.

The following study aimed to establish the ordered organization of connections within PFC. Retrograde and anterograde neuronal tracers were injected into regions of medial and lateral PFC (prelimbic (PL), VO, ventrolateral orbital (VLO) and dorsolateral orbital cortex (DLO)). We established that PFC contains extensive reciprocal connections with areas of sensory-motor cortex. Further, our findings show a clearly ordered arrangement of input and output connections and evidence for a circuit linking sensory-motor cortex with PFC.

## Material and methods

### Animals

Data was collected from 14 male CD rats (314–358 g, Charles River, UK). Animal procedures were carried out in accordance with the UK Animals scientific procedures act (1986), EU directive 2010/63 and were approved by the Nottingham Trent University Animal Welfare and Ethical Review Body. On receipt the animals were examined for signs of ill-health or injury. The animals were acclimatized for 10 days during which time their health status was assessed. Prior to surgery the animals were housed together in individually ventilated cages (IVC; Techniplast double decker Greenline rat cages). The animals were allowed free access to food and water. Mains drinking water was supplied from polycarbonate bottles attached to the cage. The diet and drinking water were considered not to contain any contaminant at a level that might have affected the purpose or integrity of the study. Bedding was supplied by IPS Product Supplies Ltd in form of 8/10 corncob. Environmental enrichment was provided in the form of wooden chew blocks and cardboard fun tunnels (Datesand Ltd., Cheshire, UK). Post-surgery the animals were individually housed in the same conditions. The animals were housed in a single air-conditioned room within the Biological support facilities barrier unit. The rate of air exchange was at least fifteen air changes per hour and the low intensity fluorescent lighting was controlled to give 12 h continuous light and 12 h darkness. The temperature and relative humidity controls were set to achieve target values of 21 ± 2°C and 55 ± 15% respectively.

Individual bodyweights were recorded on Day - 10 (prior to the start of dosing) and daily thereafter. All animals were examined for overt signs of ill-health or behavioral change immediately prior to surgery dosing, during surgery and the period following surgery. There were no observed clinical signs/symptoms of toxicity or infection. There was no significant effect on body weight development detected.

### Surgical procedures

Rats were anesthetized with isoflurane (Merial, Harlow, UK) and placed in a stereotaxic frame with the incisor bar set so as to achieve a flat skull. Buprenorphine (0.05 mg/kg i.m/s.c) and Meloxicam (up to 1 mg/kg s.c/orally) analgesia were provided peri-operatively and for several days post-operatively. Body temperature was monitored during and immediately after surgery using a rectal thermometer. Craniotomies (<1 mm) were made at predetermined stereotaxic coordinates. Sterile tracer solution was deposited into the PFC via a 0.5 μl neuro-syringe (Hamilton, Germany). Injections of anterograde (biotinylated dextran amines, BDA; Fluorescein [SP-1130] and Texas red [SP-1140], Vector laboratories CA) and retrograde tracer (4% Fluoro-Gold in distilled water, Fluorochrome, Denver, Colorado) were made into the PL, VO, VLO or DLO (100 nl/min, 2 min diffusion time), with the intention of revealing the anatomical connections of prefrontal regions. The distance between craniotomy co-ordinates (1 mm) was based on the measured spread of tracers in preliminary and the present studies (<1 mm in diameter). Injections were made at AP 3.7 mm from Bregma (A) ML 1.2 mm, 2.4 mm below cortical surface, (B) ML 1.2 mm, 3.2 mm below cortical surface, (C) ML 2.2 mm, 3.2 mm below cortical surface (D) ML 3.2 mm, 3.2 mm below cortical surface. The injection needle was positioned vertically for all tracer injections. Tracer injections were excluded (1 injection) from the study where the injection site was found to be in a different co-ordinate to that which was intended.

**Figure 1:**
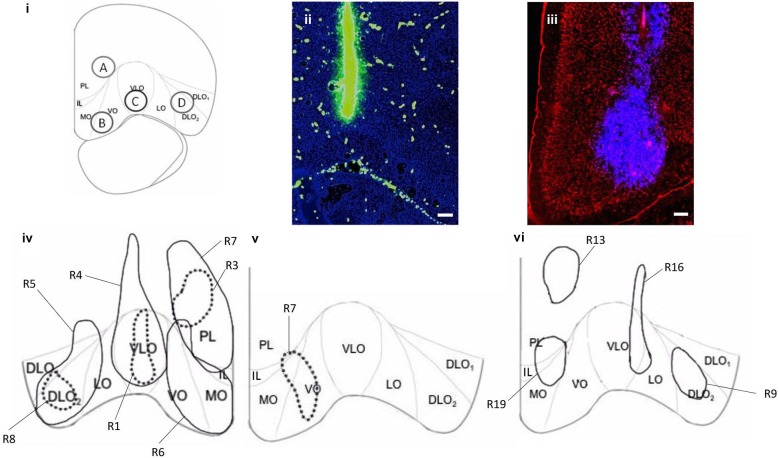
**(i)** Coronal section of PFC (AP 4.2 mm from Bregma) showing the cytoarchitectural boundaries of the prelimbic (PL), medial orbital (MO), ventral orbital (VO), ventrolateral orbital (VLO), lateral orbital (LO) and dorsolateral orbital (DLO_1_, DLO_2_) cortices (according to Van De Werd and Uylings, 2008), depicting sites of injections; Premlimbic: A, Ventral Orbital: B, Ventrolateral Orbital: C and Dorsal Lateral Orbital: D, with 1 mm spread. **(ii)** Coronal section of PFC showing location and spread of (100 nl) fluorescein (green) at injection site in VLO. **(iii)** Coronal section of PFC showing location and spread of (100 nl) Fluoro-Gold (blue) at injection site in VO/MO. **(iv)** Representations of Fluoro-Gold (100 nl) (R4, R5, R6, R7 (solid line)) and BDA (100nl) (R1, R3, R8 (broken line)) injection sites in DLO (R5, R8), VLO (R1, R4), VO/MO (R6) and PL (R3, R7), in the right hemisphere. **(v)** Representation of BDA injection site (R7) in VO/MO, in the left hemisphere. BDA injection sites were consistently within the boundaries of corresponding Fluoro-Gold injection sites. There is minimal overlap between Fluoro-Gold injection sites (R4 and R5). Fluoro-Gold injection sites are mostly limited to the cytoarchitectural boundaries of PFC regions, and span the whole region (PL, VO/MO, VLO, DLO), injections into VO spread into MO. BDA injection sites are consistently within cytoarchitectural boundaries and span layers II–VI (PL, VO/MO, VLO, DLO). **(vi)** Representations of four comparative Fluoro-Gold injection sites in PL (R13), PL/IL/MO (R19), VLO/LO (R16) and DLO_2_ (R9) in the left hemisphere, used to ascertain whether hemispheric differences affected projections. R13 is positioned higher than the corresponding right hemisphere PL injection (R7), however the majority of the injection site is confined to PL and covers the dorsal aspect of the R7 injection site. Data produced by additional FG injections **(vi)** is not included in the analysis and results section. Scale bars = 200 μm.

Each rat received injections of Fluorescein (100 nl), Texas red (100 nl) and/or Fluoro-Gold (100 nl) into various subdivisions of PFC, separated by 1 mm (Figure 1). Rats received either one injection of tracer or an injection of retrograde tracer into one hemisphere and an injection of anterograde tracer into the other hemisphere to allow accurate identification of the tracers injected. All Fluoro-Gold injections were made in the right hemisphere in this experiment (Figure 1iv) and BDA injections were also made in the right hemisphere except in the case of injection B (left hemisphere; Figures 1iv,v). Seven additional and equivalent Fluoro-Gold injections were made into the left hemisphere to verify whether the location and ordering of projections differed on the either side of the brain (four of these are shown in Figure 1vi). The same overall order and positioning of Fluoro-Gold labeling was observed on both sides of the brain.

Following a survival time of 7–8 days, the rats were deeply anesthetized with pentobarbital (Sigma-Aldrich, UK), and transcardially perfused with phosphate buffered saline (PBS) (pH 7.4) (~200 ml) followed by 4% paraformaldehyde (PFA) (pH 7.4) (~200 ml). The brain was subsequently removed and stored for 24 h in 4% PFA in PBS (pH 7.4), followed by cryoprotection in 30% sucrose in PBS.

### Anatomical processing and imaging

For analysis of anterograde connections, two series of 40 μm coronal sections were taken (two in six sections) on a freezing microtome (CM 1900, Leica, Germany). Sections were mounted onto gelatin coated slides. One series of sections was cover slipped with Vectashield® (Vector laboratories, CA) mounting medium (with DAPI), for fluorescent imaging of Fluorescein and Texas red injection sites. A second series was processed by implementing the avidin-biotin method (Vectastain® ABC, Vector laboratories), for bright field imaging of Fluorescein and Texas red labeled cells. This series of sections was counterstained with thionin. For analysis of retrograde connections, a parallel series of 40 μm coronal sections was taken (one in six sections), mounted onto gelatin coated slides, then cover slipped with Vectashield® (Vector laboratories, CA) mounting medium (with propidium iodide) for fluorescent imaging of Fluoro-Gold.

Sections were examined using either bright field (Fluorescein and Texas red) or fluorescent microscopy (Fluorescein, Texas red and Fluoro-Gold). Injection sites were determined according to the cytoarchitecture of PFC sub-regions (Van De Werd and Uylings, 2008) and labelled cells were plotted on representative coronal diagrams (Paxinos and Watson, 1998). Fluorescent photos were captured of injection sites and retrogradely labelled cells (Fluoro-Gold). Brightfield photos were captured of anterogradely labelled areas (Fluorescein and Texas red) using an Olympus DP-11 system microscope with a ×4 and ×10 objective lens.

### Microscopic analysis

Initially, the entire forebrain was examined for labeling. Areas of sensory-motor cortex were found to contain some of the strongest and most consistent labeling of connections; therefore a more detailed analysis was carried out on this region to examine the organization of prefrontal to sensory-motor cortex connections. Prominent labeling was also found in areas of temporal cortex, PFC—temporal cortex connections were investigated in a separate study (Bedwell et al., in preparation).

### Statistical analysis of the arrangement of connections between prefrontal and sensory-motor cortex

We implemented a statistical analysis to determine whether connections between PFC and sensory-motor cortex displayed an ordered arrangement. ImageJ (Wayne Rasband, NIH) was used to determine numerical values representing the three dimensional location of retrogradely labelled cells in sensory-motor cortex. The dorsoventral distance from the dorsal aspect of the cortical surface (mm) and the medial-lateral distance from the midline (mm) were measured from each retrogradely labelled cell. The anterior-posterior location of each retrogradely labelled cell was also recorded in terms of distance (mm) from Bregma (according to Paxinos and Watson, 1998). A similar acquisition of data was implemented for the anterograde data, whereby four equally spaced data points were recorded from the perimeter (i.e., at the perimeter of four quadrants) of each anterogradely labelled area of axon terminals. The dorsoventral location of each of the four data points was measured from the dorsal aspect of the cortical surface (mm) and the medial-lateral location (mm) of each data point was measured from the midline. The anterior-posterior location of each labelled area was also recorded, in terms of distance (mm) from Bregma (according to Paxinos and Watson, 1998). This resulted in four individual three-dimensional locations being recorded for each concentrated area of anterograde labeling.

Labelled cells were grouped according to injection site location. The data was analyzed in SPSS by way of a factorial ANOVA, in order to establish the existence of an effect of injection location on the positioning of labelled cells in anterior-posterior, dorsal-ventral and medial-lateral dimensions. This was followed by *post hoc* analyses of comparisons (Tukey HSD) to further investigate significant differences between four PFC injection locations. The relationship between anterograde and retrograde label locations was examined by means of a 2 way ANOVA. All statistical tests were applied at a significance level of 0.05 and confidence intervals of 95%.

## Results

Retrograde injections made into PL, VO, VLO and DLO (R4, R5, R6, R7) occurred in the intended PFC regions (Figures 1iv,v). The majority of retrograde injection sites spanned layers I–VI, covered most of the cytoarchitectural region and were principally confined to cytoarchitectural boundaries of PFC sub-regions. The retrograde injection made into VO covered both VO and MO. There was some overlap between PL and VO injection sites and some spread into infralimbic cortex (IL; Figure 1iv). Anterograde injections made into PL, VO, VLO and DLO (R1, R3, R7, R8) occurred within the same cytoarchitectural regions as the retrograde equivalents, spanning a smaller area. Anterograde injection sites were largely within the cytoarchitectural boundaries of PFC sub-regions (the injection into VO occurred in both VO and MO), covering layers II–VI (Figures 1iv,v).

**Figure 2:**
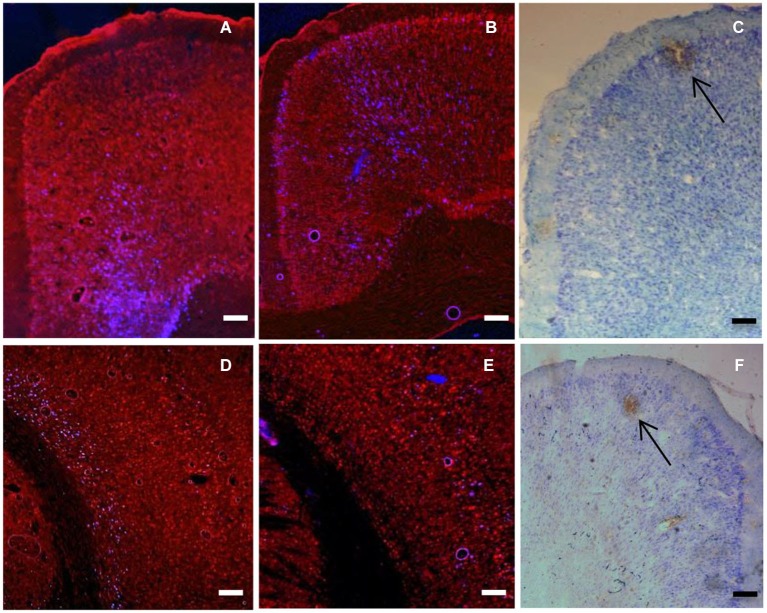
**(A)** Coronal section showing retrogradely labelled cells (blue) in cingulate cortex produced by 100 nl injection of Fluoro-gold into VLO (injection C). **(B)** Coronal section showing retrogradely labelled cells (blue) in cingulate cortex produced by 100 nl injection of Fluoro-gold into VO (injection B). **(C)** Coronal section showing anterograde labeling (brown) produced by injection of BDA (100 nl Fluorescein) into DLO (injection D). Arrow shows area of intense anterograde labeling of axon terminals. Other brown staining indicates less intense anterograde labeling, as well as some artifactual staining. Note the ordered location of labelled neurons within the dorsal-ventral axis. **(D)** Coronal section showing retrogradely labelled cells (blue) in sensory cortex produced by injections of Fluoro-Gold (100 nl) into VLO (injection C). **(E)** Coronal section showing retrogradely labelled cells (blue) in sensory cortex produced by injections of Fluoro-Gold (100 nl) into and VO (injection B). **(F)** Coronal section of sensory-motor cortex showing locations of anterograde labeling (brown) produced by injection of BDA (100 nl Texas red) into VLO (injection B). Arrow shows area of intense anterograde labeling of axon terminals. Other brown staining indicates less intense anterograde labeling, as well as some artifactual staining. Scale bars = 200 μm.

We observed the patterns of labeling throughout the brain following injections of anterograde and retrograde tracer into PFC (PL, VO, VLO and DLO) using light/fluorescent microscopy. Retrogradely labelled cells were seen in secondary motor cortex (M2), primary motor cortex (M1), primary somatosensory cortex (S1J, S1BF), cingulate cortex (Cg1), piriform cortex (Pir), perirhinal cortex (PRh), ectorhinal cortex (Ect), lateral entorhinal cortex (LEnt), secondary auditory cortex (AuV) and primary auditory cortex (Au1). Anterograde labeling was seen in M2, S1J, secondary somatosensory cortex (S2), Cg1, PRh, Ect, LEnt, agranular insular cortex (AID) and PFC regions. Labeling was stronger in several regions, including sensory-motor areas (M1, M2, S1J; Figure 2). Therefore a statistical analysis was applied to this region to determine whether there was evidence for an ordered arrangement of prefrontal to sensory-motor cortex connections.

### Organization of input and output connections from sensory-motor to prefrontal cortex

**Figure 3:**
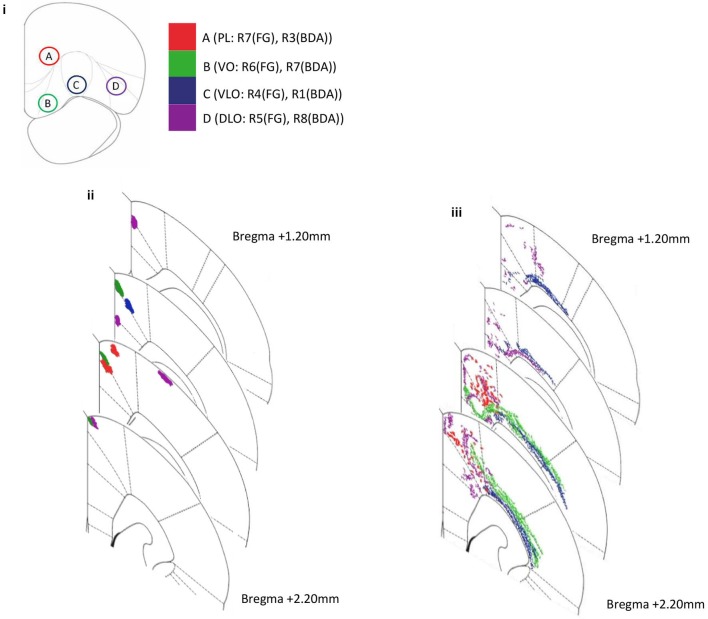
**Diagram representing amalgamated injection sites within PFC and subsequent projection sites to sensory-motor cortex for both anterograde (BDA) and retrograde (FG) tracer injections in several rats**. Coronal sections depict the injection site and projecting site. **(i)** The positions of four injection sites within PFC; PL (injection A: R7(FG), R3(BDA)), VO (injection B: R6(FG), R7(BDA)), VLO (injection C: R4(FG), R1(BDA)) and DLO (injection D: R5(FG), R8(BDA)). **(ii)** Anterograde labeling of axon terminals (PFC output connections) following injections into four PFC sites (A–D BDA: R1, R3, R7, R8). **(iii)** Retrograde labeling (PFC input connections) following injections into three PFC sites (A–D, FG: R4, R5, R6, R7). Note the ordered location of labeled areas/neurons within the dorsal-ventral and medial-lateral axes.

Retrogradely labelled cells were seen in secondary motor (M2), primary motor (M1) and primary somatosensory cortex (S1J). Labelled cells in the sensory-motor region showed an ordered arrangement in terms of cortical layers (Figure 3). Labelled cells produced by an injection of retrograde tracer into DLO appear in layer VI, whereas those produced by an injection of tracer into VLO appear in layer V in the same region. Cells labelled following an injection of tracer into VO appear across layers II to VI. In addition, retrogradely labelled cells were consistently seen in deeper cortical layers (VI, V) than anterogradely labelled areas (I, II; Figure 3).

Anterogradely labelled areas were seen in M1, M2 and cingulate cortex (cg1). Anterogradely labelled areas found in motor cortex (M1, M2) maintain a relatively clear spatial order in correspondence to BDA tracer injections in VO, VLO and DLO. As injection sites move from lateral (DLO) to medial (VO), labelled areas become more dorsal. A convergent organization can be seen here; injections into separate PFC regions (separated by 1 mm) produced labeling in the same motor region (M2). Labelled areas were physically closer to one another than their corresponding injection locations (Figure 3).

We observed evidence of reciprocal connectivity in the PFC—sensory-motor cortex pathway; anterogradely labelled output connections were consistently found in the same regions of motor cortex (M2) as retrogradely labelled input connections from identical PFC injection sites. This was the case for injections into PL, VO, VLO and DLO. Retrogradely labelled cells were found in additional regions to those in which corresponding anterograde labels were seen. The level of reciprocity appeared to be greater for PL than for VO, VLO and DLO, anterograde labeling from PL was found mostly in the same regions of M2 and Cg1 as retrograde labeling from PL.

## Statistical evidence for the ordered arrangement and location of input and output connections came from the following analyses

A factorial ANOVA was applied to the locations of retrogradely labelled cells in each axis of orientation. This was repeated for the locations of anterogradely labelled areas. A 2 factor ANOVA (injection type[anterograde, retrograde], injection location [A,B,C,D]) was applied in order to establish the relationship between input and output connections.

**Figure 4:**
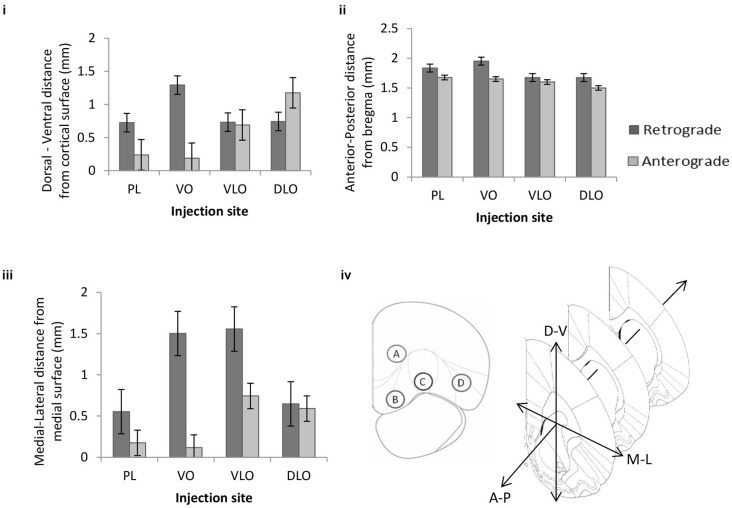
**The mean effect of injection site on the (i) dorsal-ventral, (ii) anterior-posterior and (**iii**) medial-lateral location of retrogradely cells (*n* = 494 cells arising from four rats: PL = 143, VO = 156, VLO = 83 and DLO = 112 cells) and anterogradely labelled areas (*n* = 40 points from four rats: PL = 8, VO = 12, VLO = 4 and DLO = 16 points) within the sensory-motor cortex**. Error bars = standard error. **(iv)** Coronal cross section of PFC indicating the position of four injection sites within PFC: Prelimbic (injection A), Ventral Orbital (injection B), Ventrolateral Orbital (injection C) and Dorsal Lateral Orbital (injection D), coronal cross section of sensory-motor cortex, depicting the three dimensions in which the locations of labelled cells were recorded.

**For the dorsal-ventral axis**: The factorial ANOVA revealed a significant main effect of injection site on dorsoventral (i.e., laminar) location of retrogradely (*F*_(3,490)_ = 380.578, *p* < 0.001) and anterogradely (*F*_(3,36)_ = 23.719, *p* < 0.001) labelled cells in sensory-motor cortex. *Post hoc* comparisons (Tukey HSD) between the four retrograde groups indicate significant differences between PL*VO, PL*VLO, PL*DLO (*p* < 0.001). *Post hoc* comparisons (Tukey HSD) between the four anterograde groups indicated significant differences between PL*DLO, VO*DLO (*p* < 0.001) and VLO*DLO (*p* = 0.028). This indicates an ordered arrangement from sensory-motor areas to medial sub-regions of PFC (PL, VO, VLO; Figure 4i). The 2 factor ANOVA revealed a significant interaction effect between input and output connections (*F*_(3,526)_ = 45.709, *p* < 0.001). This shows that the dorsoventral location of anterogradely and retrogradely labelled cells vary in respect to one another. The statistical analysis revealed a significant ordering of retrograde and anterograde connections. The 2 factor ANOVA indicated that the input and output connections (anterograde and retrograde label) occurred in different locations in this axis of orientation.

**For the anterior-posterior axis**: A factorial ANOVA revealed a significant main effect of injection site on anterior-posterior location of retrogradely labelled cells (*F*_(3,490)_ = 82.090, *p* < 0.001) and anterogradely labelled cells (*F*_(3,36)_ = 6.029, *p* = 0.002) in sensory-motor cortex. *Post hoc* comparisons (Tukey HSD) between the four retrograde groups indicated significant differences between PL*VO, PL*VLO, PL*DLO, VO*VLO, VO*DLO (*p* < 0.001). *Post hoc* comparisons (Tukey HSD) between the four anterograde groups indicated significant differences between PL*DLO and VO*DLO (*p* < 0.006). The 2 factor ANOVA revealed no significant interaction effect between input and output connections (*F*_(3,526)_ = 2.415, *p* = 0.066). This indicates an ordered arrangement of input and output connections within the anterior-posterior axis (Figure 4ii). This analysis shows strong evidence for ordering of anterograde connections as well as evidence for ordering of retrograde connections. There is no clear statistical evidence for differential location of inputs and outputs in this axis of orientation.

**For the medial-lateral axis**: A factorial ANOVA revealed a significant main effect of injection site on medial-lateral location of retrogradely (*F*_(3,490)_ = 385.767, *p* < 0.001) and anterogradely (*F*_(3,36)_ = 24.102, *p* < 0.001) labelled cells in sensory-motor cortex. *Post hoc* comparisons (Tukey HSD) between the four retrograde groups indicated significant differences between PL*VO, PL*VLO, PL*DLO (*p* < 0.001). *Post hoc* comparisons (Tukey HSD) between the four anterograde groups indicated significant differences between all injection sites; PL*VLO, PL*DLO, VO*VLO and VO*DLO (*p* < 0.001). Figure 4iii shows that no clear ordered arrangement is seen in the medial-lateral axis. The 2 factor ANOVA revealed a significant interaction effect between input and output connections (*F*_(3,526)_ = 24.695,* p* < 0.001). This shows that the medial-lateral location of anterogradely and retrogradely labelled cells vary in relation to one another. The statistical analysis revealed a significant ordering of retrograde and anterograde connections. The 2 factor ANOVA indicated that the input and output connections (anterograde and retrograde label) occurred in different locations in this axis of orientation. Figure 4iv summarizes the approximate position of injection sites and shows the orientations shown in Figures 4i–iii.

## Discussion

We investigated the organization of connections from adjacent prefrontal regions with the use of neuroanatomical tracers. This study revealed evidence for an ordered arrangement within the projections from PFC to sensory-motor cortex. We revealed a clear ordered arrangement of PFC connections, most prominently arising from significant portions of sensory-motor cortex. In addition we found evidence of a differential ordering of input and output locations between PFC and sensory-motor regions, in the dorsoventral and mediolateral axes.

### Input connections from sensory-motor cortex to prefrontal cortex

Following the administration of the retrograde tracer, Fluoro-Gold, to the prefrontal cortical areas PL, VO, VLO and DLO, we found labeling of neuronal cell bodies in areas of motor cortex, temporal cortex, auditory cortex, somatosensory cortex, cingulate cortex and piriform cortex. Projections arising from the motor cortex were amongst those displaying the most labelled cells. These findings are comparable with previous studies (Sesack et al., 1989) which outlined projections from prefrontal to areas of sensory-motor cortex in the rat.

### Output connections from sensory-motor cortex to sensory-motor cortex

Following the administration of the anterograde tracers, BDA to the prefrontal cortical areas PL, VO, VLO and, DLO we found labeling of axon terminals in motor cortex, temporal cortex, auditory cortex, somatosensory cortex, agranular insular cortex, cingulate cortex and PFC regions. The projections arising from the motor and cingulate cortex (M1, M2 and cg1) were amongst those displaying the most labeling. This was broadly consistent with previous studies which outlined prefrontal connections to medial-frontal cortex (Sesack et al., 1989; Vertes, 2004), in that anterograde labels were found in the same regions of cingulate, motor and somatosensory cortex in the rat.

### The organization of connections between prefrontal and sensory-motor cortex cortex

Our analysis of the location of input and output connections to and from prefrontal regions shows an ordered arrangement of connections occurring in and across three axes of orientation, most significantly so in the anterior-posterior and medial-lateral axes. The analysis of sensory-motor cortex connections revealed an ordered arrangement of input and output connections to PFC occurring in the dorsal-ventral (i.e., laminar) axis (Figure 4i). For the dorsal-ventral axis, injections in sites B (VO), C (VLO) and D (DLO) produced an ordered arrangement of PFC output connections to sensory-motor cortex. There was some striking ordering in terms of the input connections from sensory-motor cortex (notably M1 and S1) to PFC. Here specific layers of V and VI differentially project to injection sites B (VO) and C (VLO). Similar laminar organization has been described elsewhere in the same or neighboring circuit: projections from rat medial PFC to striatum, amygdala and thalamus occur in distinct layers of mPFC (Gabbott et al., 2005). This may be of particular significance to PFC function because both of these regions (M1 and S1) contain somatotopic maps. It appears that the same regions implicated in these maps (in layers V and VI) project to distinct regions of PFC (i.e., VO and VLO). It could be functionally useful for the same ordered arrangement to be maintained within sensory-motor to PFC connections.

Our results show a projection from M1, M2 and S1 to PFC and on to M2. This circuit is not typical of the connectional organization we might expect to see. Based on a traditional model of cortical function and hierarchical organization of functional connectivity, PFC is positioned at the top of a processing hierarchy (Fuster, 2001; Botvinick, 2008). Subsequently connections would travel from primary sensory cortex, followed by secondary sensory cortex and association areas such as perirhinal cortex before reaching PFC. Finally, return connections would travel to secondary motor cortex (M2) followed by primary motor cortex (e.g., S1→ S2 → Association areas → PFC → M2 → M1), following an order of hierarchical organization from primary cortical regions through secondary regions up to a high order area, then back through secondary regions to a primary region. The connections we have identified from PFC → M2 and M2 → PFC are consistent with what would be expected, based on the model of hierarchical organization. However, the direct connection from S1→PFC we have observed is unexpected, connecting a primary cortical region directly to the high order PFC. This provides an insight into the complex functional organization of the PFC—sensory-motor cortex pathway.

Our findings show evidence of reciprocal connections between PFC and M2. Anterogradely labelled axon terminals and retrogradely labelled neuronal cells from identical PFC injection sites were consistently found in the same cytoarchitectural regions of M2. This indicates a broad reciprocal organization. However, labeling from retrograde injections was considerably more widespread than that from anterograde injections, resulting in retrograde labels in areas of M1 and S1J. No anterograde labels were seen in these regions, resulting in connections which are not reciprocal. Labeling from PL (injection site A) was found to be more reciprocal than VO, VLO and DLO. These results show that the PFC—sensory-motor cortex pathways contain aspects of reciprocity but is not entirely reciprocal in its organization.

The current findings may be of significance for functional studies of the prefrontal and motor cortex. Prefrontal cortex has long been known to have important inputs to premotor and motor cortices. Recent studies have investigated the functional similarity of medial PFC and motor cortex in the coding of temporal aspects of motor behavior. Neurons in medial PFC and motor cortex display modulated activity during simple reaction time tasks (Narayanan and Laubach, 2009) and neuronal responses in dorsal mPFC are also modulated following errors in similar tasks (Narayanan and Laubach, 2008). Such functional studies highlight the connectional links between PFC and motor cortex, a similar functional study has shown that motor cortex delay-related activity is dependent upon activity within mPFC (Narayanan and Laubach, 2006). In the present study our findings add another level of complexity to this area by highlighting the connections of orbital cortex to motor cortex and the significant, indirect input of somatosensory cortex to motor cortex (via the orbital PFC).

### Significance of ordered arrangements and differential ordering of input and output connections from PFC to sensory-motor cortex

The results presented here outline that PFC displays an ordered arrangement of connections to sensory-motor cortex. The input connections to PFC arise from distinct regions of sensory-motor cortex, notably different layers within motor and S1 somatosensory cortex. In terms of outputs from PFC the projections are to distinct regions of sensory-motor cortex, notably M2 and M1. Therefore there appears to be a broad similarity in location between PFC inputs and outputs to sensory-motor cortex. However, at a more detailed level there is a separation in the location of inputs and outputs to PFC. Labelled input connections can be seen spreading significantly more laterally into regions of motor (M1) and sensory (S1J) cortex in comparison to the consistently medial (Cg1, M2) output labels (Figure 3). This provides clear evidence of different locations of input and output connections revealed by differential ordering of anterograde and retrograde labels. Such a differential ordering of inputs and outputs has not been previously described in PFC. A similar differential ordering of input and output connections was also seen in our study of the temporal cortex—PFC pathway (Bedwell et al., in preparation). Previous observations in organization suggest reciprocity of inputs and outputs to be a common attribute in perirhinal, postrhinal, entorhinal and parahippocampal regions in rats (Agster and Burwell, 2009). In addition, recent dual tracer studies have given no indication that the locations of input and output connections to ventrolateral and medial PFC from premotor cortex, as well as subcortical regions, differ (Kim and Lee, 2012). However, these findings report PFC connectivity on a relatively large scale and from specific sub-regions.

Few studies of rodent PFC have addressed the subject of whether finescale input and output connections occupy the same location (Agster and Burwell, 2009). However, connectional studies of primary sensory (S1) and primary motor cortex (M1) often report reciprocity of connections (Dinopoulos, 1994; Lee et al., 2011). Further, detailed studies of somatosensory and motor cortex in rodent species have shown that inputs and outputs occupy the same, precise locations in the connections between S1 and S2 (Henry and Catania, 2006; Aronoff et al., 2010), M1 and S1 (Porter and White, 1983; Aronoff et al., 2010) and between M1 and S2 (Porter and White, 1983). The study by Porter and White also reported non-reciprocal connections from M1 to the striatum, indicating that motor cortex input and output connections do not always occupy the same location.

An interesting aspect of our results is the finding that the input and output connections from Prelimbic cortex to Cg1 and M2 are largely reciprocal and occur in the same locations (if laminar differences are ignored). This is a very different pattern to that produced after the other injections (notably VO and VLO) which existed in different locations. The significance of this difference in the finescale reciprocity of connections across PFC divisions is not clear but it may relate to the function and organization of the connected regions involved.

Taken together our results indicate that the prefrontal to sensory-motor cortex inputs and outputs display differential ordering and occupy different locations, this differs to the organization displayed between visual and sensorimotor cortical areas (where inputs and outputs display the same ordering and exist in the same locations). It is too early to say whether this differential ordering of input and output locations is a common feature of PFC connections. However, in our laboratory we have found evidence for it in two PFC pathways, i.e., between PFC and sensory-motor cortex (presented here) and between PFC and the temporal cortex.

## Conflict of interest statement

The authors declare that the research was conducted in the absence of any commercial or financial relationships that could be construed as a potential conflict of interest.
